# Correlation of Transcutaneous pCO_2_ and Capillary pCO_2_ in Newborn Patients Depending on Gestational Age: A Secondary Data Analysis

**DOI:** 10.1002/ppul.71733

**Published:** 2026-07-12

**Authors:** Lena Olivier, Camelia Lauterbach Oprea, André Stollenwerk, Valerie Pfannschmidt, Thorsten Orlikowsky, Mark Schoberer

**Affiliations:** ^1^ Division of Neonatology, Department of Pediatric and Adolescent Medicine Uniklinik RWTH Aachen Aachen Germany; ^2^ Chair of Embedded Software (Computer Science 11) RWTH Aachen University Aachen Germany

**Keywords:** carbon dioxide, neonatal intensive care unit, neonate, premature infants, transcutaneous blood gas monitoring, transcutaneous capnometry

## Abstract

**Introduction:**

Transcutaneous monitoring of CO_2_ partial pressure (tcpCO_2_) provides continuous information about the CO_2_ partial pressure in the blood (pCO_2_). However, the deviation of tcpCO_2_ from pCO_2_ is variable. Our aim was to assess whether this deviation depends on gestational age in the first postnatal week.

**Methods:**

Single‐center secondary data cohort study at a Tertiary Care NICU. 116 neonates undergoing tcpCO_2_ monitoring were included. A linear mixed‐effects model was used to analyze the difference between tcpCO_2_ and capillary pCO_2_ (ΔpCO_2_) in the first postnatal week.

**Results:**

926 simultaneous measurements were included. The evaluation of the linear mixed‐effects model revealed a significant effect of gestational age on ΔpCO_2_. Median [interquartile 1; interquartile 3] ΔpCO_2_ was significantly higher for extremely preterm infants (< 28 weeks) with 7.6 [2.4; 13.9] mmHg compared to 3.1 [−1.0; 6.4] mmHg for very preterm (28−31 + 6 weeks), 0.6 [−4.6; 4.7] mmHg for late preterm (32−36 + 6 weeks) and −0.5 [−4.8; 4.7] mmHg for term infants (≥ 37 weeks). There were no significant differences in ΔpCO_2_ between patients born at 23 to 25 compared to 26 and 27 completed weeks of gestation. In multivariable analysis, gestational age, early‐onset bacterial infection and capillary pCO_2_ were independently associated with ΔpCO_2_, whereas birth weight was not.

**Conclusions:**

The gestational age has a significant directed effect on the accuracy of transcutaneous CO_2_ monitoring in preterm neonates and can—together with other known influence factors—be used to improve the non‐invasive pCO_2_ estimation.

**Abbreviations**
EOBI: early‐onset bacterial infection
EPI: extremely preterm infants (< 28 weeks)
LMM: linear mixed‐effects model
LPI: late preterm infants (32 + 0 to 36 + 6 weeks)
NICU: neonatal intensive care unit
paCO_2_
: arterial partial pressure of carbon dioxide
pcCO_2_
: capillary partial pressure of carbon dioxide
pCO_2_
: partial pressure of CO_2_
tcpCO_2_
: transcutaneous partial pressure of carbon dioxide
TI: term infant (≥ 37 weeks)
VPI: very preterm infant (28 + 0 to 31 + 6 weeks)
ΔpCO_2_
: difference between tcpCO_2_ and pcCO_2_

## 1 Introduction

### 1.1 Background

About 10% of neonates are admitted to a neonatal intensive care unit (NICU) [1, 2]. A large proportion of these infants suffer from respiratory distress or failure and require respiratory support [3, 4]. Among others, prematurity, surfactant deficiency, and sepsis can cause pulmonary failure [5]. Treatment options comprise non‐invasive or invasive ventilatory support to ensure sufficient gas exchange [6].

During assisted ventilation, it is essential to avoid hypo‐ and hypercarbia, as the arterial partial pressure of carbon dioxide (paCO_2_) influences the cerebral perfusion [7]. While hypocarbia can lead to hypoxia of the brain tissue and entail periventricular leucomalacia [8], severe hypercarbia and fluctuations of paCO_2_ [9, 10] may increase the cerebral blood flow and hereby contribute to the pathogenesis of intraventricular hemorrhage [11]. Both conditions can dysregulate the acid‐base balance and bring about serious long‐term neurodevelopmental impairment [12, 13]. The close monitoring of blood gases is fundamental to prevent and recognize extremes of paCO_2_ and to adapt treatment if necessary. This monitoring is typically conducted by blood gas analyses. Blood sampling can only provide discontinuous measurements and causes pain, for example, if blood samples are taken from capillaries. Furthermore, it may lead to a substantial loss of blood, which was found to be associated with the need for blood transfusions [14], and which can in turn contribute to the development of bronchopulmonary dysplasia [15].

Therefore, non‐invasive paCO_2_ monitoring techniques are frequently used to gain continuous information about the elimination of CO_2_ and to reduce the number of blood gas analyses. In this context, NICUs often use transcutaneous measurements of CO_2_ partial pressure (tcpCO_2_) [16]. For tcpCO_2_ monitoring, a sensor is applied to the skin. It heats up to a constant temperature to ensure a sufficient capillary blood flow and to enhance the diffusion of CO_2_ from the capillary blood through the skin to the sensor [17]. The probe then quantifies the CO_2_ partial pressure at the skin surface. The use of tcpCO_2_ monitoring can reduce the number of conducted blood gas analyses [18] and is therefore compatible with minimal handling strategies [19].

However, literature about the accuracy of tcpCO_2_ measurements is inconsistent [20]. Among others, body weight, age, tcpCO_2_ values, and ranges of the blood partial pressure of CO_2_ (pCO_2_) are known to confound the estimation of pCO_2_ from tcpCO_2_ in neonates [21, 22]. Most researchers report a good correlation of tcpCO_2_ and pCO_2_ for neonatal, pediatric and adult intensive care patients [21, 22, 23, 24, 25]. In contrast, other research groups only found a moderate [18, 26] or poor agreement of tcpCO_2_ and pCO_2_ trends, for example, in a cohort of very premature infants [27].

### 1.2 Objective

We hypothesized that the gestational age at birth influences the accuracy of tcpCO_2_ monitoring in the first postnatal week.

## 2 Materials and Methods

### 2.1 Study Design

The analysis was conducted as a secondary data cohort study. Data were collected in the AIx‐Neo‐Guard project, a collaborative project of the NICU of University Hospital RWTH Aachen, Germany, and the Chair of Embedded Software of RWTH Aachen, Germany. The project's objective is to apply secondary data analyses (including artificial intelligence‐based approaches) to address health‐related aspects of neonatal and pediatric intensive care treatment. The legal framework for data collection was provided by the German Health Data Utilization Act. The Health Data Utilization Act granted an exemption from parental informed consent, as only data collected for diagnostic and therapeutic purposes were used, with no additional data being gathered. Data have been collected since August 2024. The database incorporates time‐series data from 357 pediatric patients of all age groups (as of November 30, 2025). The overall study and exemption from informed consent were approved by the local ethics committee (Ethik‐Kommission an der Medizinischen Fakultät der Rheinisch‐Westfälischen Technischen Hochschule Aachen (RWTH Aachen), approval number EK 24‐213). The study is in accordance with the Declaration of Helsinki [28] and the “Strengthening the Reporting of Observational Studies in Epidemiology” statement by von Elm et al. [29].

### 2.2 Setting

Data were collected at the Tertiary Care NICU of University Hospital RWTH Aachen, Germany.

### 2.3 Participants

All newborn patients of all gestational ages treated at the NICU of University Hospital RWTH Aachen between August 2024 and November 2025 were considered eligible. Patients were included if they were monitored by both blood gas analyses and a transcutaneous CO_2_ sensor for respiratory considerations in their first postnatal week. Patients were excluded if parents objected to study participation, if they underwent therapeutic hypothermia, or if there were alterations of the skin which might impact transcutaneous monitoring (including generalized congenital dermatosis, infections of the skin, and anasarca). We furthermore excluded infants with periods of hemodynamic shock, defined as persistent arterial hypotension (mean arterial pressure < gestational age) with the need for vasopressor therapy.

### 2.4 Variables

Patient characteristics used for analysis were gestational age, postnatal age, birth weight, birth weight percentile and early‐onset bacterial infections (EOBI, defined as clinical impairment and elevation of infection parameters in the blood). Measurement variables included tcpCO_2_ and capillary pCO_2_ (pcCO_2_). Further patient characteristics are presented in Table 1.

**Table 1:** Group characteristics (shown as mean ± standard deviation).

	EPI	VPI	LPI	TI	Total
Number of patients (*n*)	22	26	39	29	116
Number of measurements (*n*)	346	282	181	117	926
Gestational age (weeks)	25.5 ± 1.4	29.5 ± 1.3	34.2 ± 1.6	38.6 ± 1.4	32.6 ± 4.9
Birth weight (g)	781 ± 183	1437 ± 347	2229 ± 668	3334 ± 388	2053 ± 1018
Birth weight percentile	33 ± 17	50 ± 24	34 ± 27	48 ± 26	41 ± 25
Male sex (%)	54.5	53.8	51.2	48.3	51.7
5 min APGAR	8 ± 1	8 ± 1	8 ± 1	8 ± 2	8 ± 1
Umbilical cord pH	7.28 ± 0.11	7.30 ± 0.06	7.28 ± 0.08	7.21 ± 0.11	7.27 ± 0.1
Born by C‐section (%)	100	96	79	59	83
Early‐onset bacterial infection (%)	27	12	13	34	21
Mechanical ventilation (%)	45	15	8	14	18

### 2.5 Data Sources and Measurement

Patient characteristics were abstracted from the local patient data management system. pcCO_2_ was taken from capillary blood gas analyses, which were measured with an ABL90 FLEX Plus analyzer (Radiometer GmbH, Krefeld, Germany). tcpCO_2_ measurement was conducted with a Sentec Digital Monitor with V‐Sign sensor (SENTEC AG, Therwil, Switzerland). The sensor was applied to the patients' chest, thigh, or back and heated up to a temperature of 40.2°C. Re‐calibration was conducted during nursing, when routinely required by the monitor algorithm or if the correlation was assessed to be poor by the medical staff. We took the corresponding tcpCO_2_ value, which was recorded closest to the time of blood gas analysis within the window of tolerance (±5 min). The difference between tcpCO_2_ and pcCO_2_ measurement (ΔpCO_2_) was calculated by subtracting pcCO_2_ from tcpCO_2_.

### 2.6 Bias

To evade selection bias, we included all eligible newborn patients. Poor agreement of tcpCO_2_ and pcCO_2_ did not lead to an early termination of tcpCO_2_ monitoring. All neonates were treated according to local standards. tcpCO_2_ and pcCO_2_ measurements were conducted by the same type of measuring devices, respectively in all patients to minimize detection bias.

### 2.7 Study Size

We included all eligible patients with tcpCO_2_ and pcCO_2_ monitoring in their first postnatal week.

### 2.8 Quantitative Variables

Gestational age was categorized as extremely preterm (< 28 weeks, EPI), very preterm (28 + 0 to 31 + 6 weeks, VPI), moderate to late preterm (32 + 0 to 36 + 6 weeks, LPI), and term (≥37 weeks, TI), according to the definition of the World Health Organization [30].

### 2.9 Statistical Methods

We used linear mixed‐effects models (LMM) to analyze the effect of gestational age, EOBI, birth weight, and pcCO_2_ on ΔpCO_2_. The LMM was chosen as each patient contributed a variable number of repeated measurements (1−39 measurements per patient). The respective univariate model included the categorized gestational age, EOBI, birth weight, or pcCO_2_ as a fixed effect and a random intercept for each participant. The model was fitted using restricted maximum likelihood. Fixed effects were evaluated by Type III *F*‐tests with Satterthwaite‐adjusted degrees of freedom. Variance components were estimated for both the subject‐level random intercept and the residual error term. The intraclass correlation coefficient was calculated to quantify the proportion of total variance which contributes to between‐subject differences. Effect size estimates were reported using marginal and conditional R^2^ values for mixed models. Post hoc pairwise comparisons were conducted using Bonferroni analysis.

In addition to the univariate analyses, a multivariable LMM was constructed to assess whether the categorized gestational age was independently associated with ΔpCO_2_ after adjustment for potential confounding variables. The multivariable model included gestational age, EOBI, birth weight, and pcCO_2_ simultaneously as fixed effects and a random intercept for each participant to account for repeated measurements within individuals.

As the group of EPI is very heterogeneous, another LMM was used for stratification and comparison of the most immature infants of 23–25 weeks gestational age to the group of 26–27 weeks.

pcCO_2_ measurements without simultaneous measurement of tcpCO_2_ were excluded from the analysis. Percentage similarity (PS) values were calculated for each group according to Scott et al. [31], as shown below:

PS=100×(((tcpCO2+pcCO2)/2)/pcCO2)

Furthermore, a Pearson correlation analysis was conducted. Statistical significance was defined as *p* < 0.05. All analyses were performed using SPSS, version 29.0.0.0 (IBM, Armonk, USA).

## 3 Results

### 3.1 Patient Characteristics

**Figure 1:**
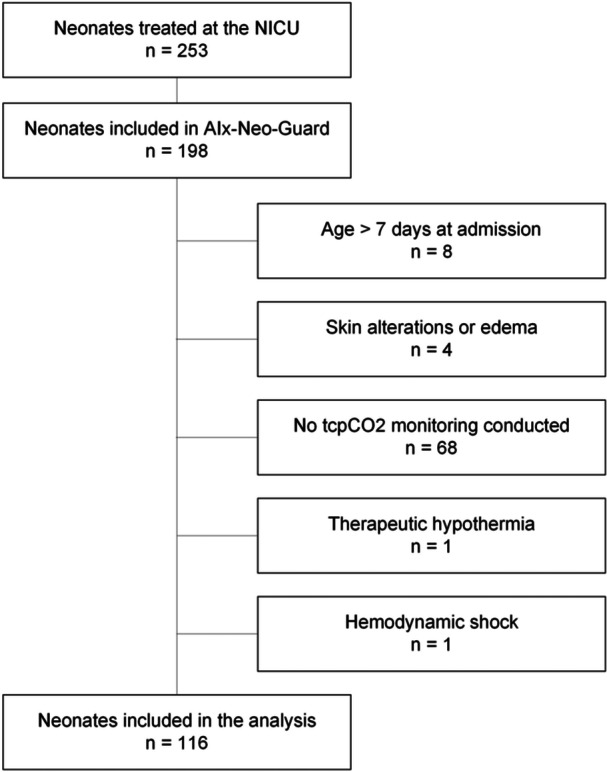
Study population flow chart.

During the study period, 253 neonates were treated at the NICU. Fifty‐five of these were not included in the AIx‐Neo‐Guard project due to technical aspects. 116 of 198 neonates met the inclusion criteria and were included in the analysis (Figure 1). Group characteristics are shown in Table 1.

### 3.2 Main Results

A total of 926 simultaneous tcpCO_2_ and pcCO_2_ measurements were analyzed. Median [interquartile 1; interquartile 3] of ΔpCO_2_ was 7.6 [2.4; 13.9] mmHg for EPI, 3.1 [−1.0; 6.4] mmHg for VPI, 0.6 [−4.6; 4.7] mmHg for LPI, and −0.5 [−4.8; 4.7] mmHg for TI.

The LMM revealed a significant effect of the patient group according to gestational age on ΔpCO_2_ (*F*(3, 109.779) = 13.910, *p* < 0.001). Compared to the reference group (TI), both EPI and VPI showed a significantly larger positive estimate (EPI: 8.42, 95% CI [5.24, 11.61], *p* < 0.001, VPI: 3.82, 95% CI [0.69, 6.95], *p* = 0.017). In contrast, the estimate of LPI did not differ from TI (0.55, 95% CI [−2.58, 3.68], *p* = 0.729). Post hoc pairwise comparisons demonstrated that ΔpCO_2_ in EPI differed significantly from ΔpCO_2_ in the other three groups, while there were no statistically significant differences in ΔpCO_2_ among the other groups.

The intraclass correlation coefficient (ICC = 0.163) indicated that approximately 16% of the total variance in ΔpCO2 was attributable to between‐subject differences, supporting the use of a mixed model. The marginal and conditional R^2^ values were 0.112 and 0.257, respectively, indicating that the fixed effects explained 11% of the variance, and the full model including random effects explained 26% of the variance.

pcCO_2_ levels did not differ significantly between the four groups (*F*(3, 922) = 0.299, *p* = 0.926). Median overall pcCO_2_ was 46.4 [40.7, 53.4] mmHg. For EPI, median pcCO_2_ was 46.4 [40.2; 53,8] mmHg, 45.9 [40.8; 52.9] mmHg for VPI, 47.1 [41.2; 52.6] mmHg for LPI, and 46.7 [40.9; 54.1] for TI. Linear mixed‐effects modeling demonstrated a significant association between pcCO_2_ and ΔpCO_2_ (*F*(1, 809) = 159.75, *p* < 0.001), indicating that the agreement between tcpCO_2_ and pcCO_2_ varied across the pCO_2_ range.

ΔpCO_2_ was also significantly associated with EOBI (*F*(1, 924) = 26.889, *p* < 0.001). EOBI patients exhibited significantly higher ΔpCO_2_ (6.5 [1.3; 11.8] mmHg) compared to non‐EOBi patients (2.9 [−1.8; 7.8] mmHg).

There was also a significant association between birth weight and ΔpCO_2_ (*F*(1, 923) = 73.01, *p* < 0.001), with lower birth weight being associated with higher ΔpCO_2_.

Since gestational age group, EOBI, birth weight, and pcCO_2_ were all significantly associated with ΔpCO_2_ in the univariate analyses, their independent contributions were assessed in a multivariable LMM. Gestational age (*F*(3, 918) = 14.47, *p* < 0.001), pcCO_2_ (*F*(1, 918) = 8.84, *p* = 0.003), and EOBI (*F*(1, 918) = 24.43, *p* < 0.001) remained independently associated with ΔpCO_2_, whereas birth weight was no longer significantly associated with ΔpCO_2_ (*p* = 0.16).

For the subgroup of EPI, there was no significant difference between the group of 23−25 weeks (*n* = 8) and 26−27 weeks (*n* = 14, *F*(1, 19.732) = 3.103, *p* = 0.094).

**Figure 2:**
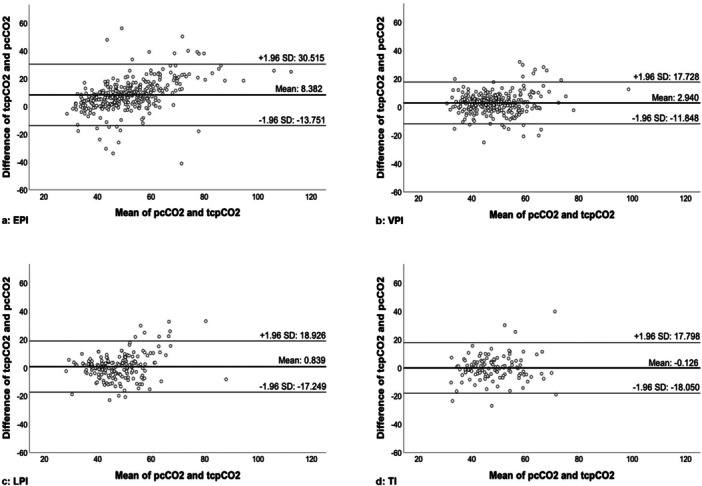
(a–d) Bland‐Altman plots with limits of agreement for each group.

**Figure 3:**
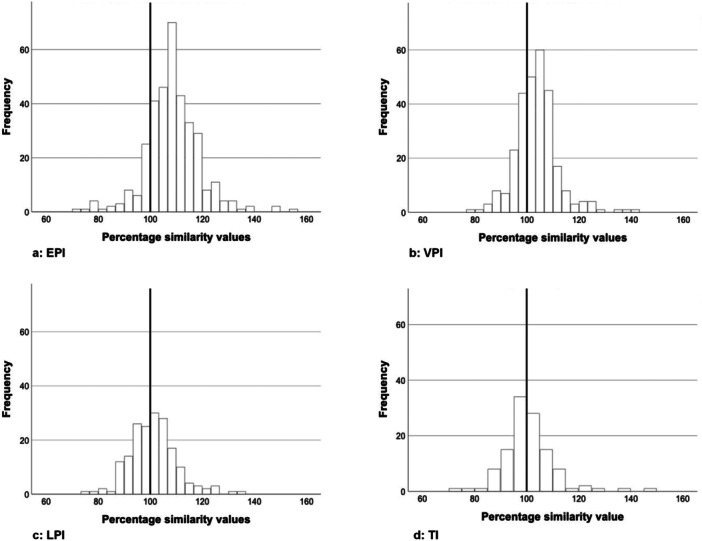
(a–d) Relative frequency distribution (%) of percentage similarity (PS) values between tcpCO_2_ and pcCO_2_ measurements. PS is calculated as the average estimate of tcpCO_2_ and pcCO_2_ relative to pcCO_2_. The vertical line indicates the 100% similarity reference value, corresponding to perfect agreement between tcpCO_2_ and pcCO_2_. Values to the right of the line (> 100%) indicate higher tcpCO_2_ than pcCO_2_ values, whereas values to the left (< 100%) indicate lower tcpCO_2_ values. PS values were grouped into 30 equal 5% intervals and plotted against relative frequency on the y‐axis.

Bland‐Altman plots with the limits of agreement for tcpCO2 and pcCO2 measurements are provided in Figure 2 and PS histograms in Figure 3 for each group.

### 3.3 Other Analyses

The results of the Pearson correlation analysis are shown in Table 2. The overall correlation of tcpCO_2_ and pcCO_2_ was moderate (*r* = 0.671). ΔpCO_2_ correlated moderately with tcpCO_2_ values (*r* = 0.658). There was a weak correlation between ΔpCO_2_ and postnatal age and birth weight (*r* = 0.33 and *r* = −0.271), but no correlation with birth weight percentile.

**Table 2:** Pearson correlation analysis.

		pcCO_2_ (mmHg)	tcpCO_2_ (mmHg)	∆pCO_2_ (mmHg)	Birth weight (g)	Weight percentile	Postnatal age (days)
pcCO_2_ (mmHg)	Pearson correlation	1	0.671**	−0.089**	0.074*	0.080*	−0.027
	Sig. (2‐tailed)		< 0.001	0.007	0.024	0.015	0.405
	*N*	926	926	926	926	926	926
tcpCO_2_ (mmHg)	Pearson correlation	0.671**	1	0.658**	−0.150**	0.059	0.215
	Sig. (2‐tailed)	< 0.001		< 0.001	< 0.001	0.071	< 0.001
	*N*	926	926	926	926	926	926
∆pCO_2_ (mmHg)	Pearson correlation	−0.089**	0.658**	1	−0.271**	−0.001	0.330
	Sig. (2‐tailed)	0.007	< 0.001		< 0.001	0.974	< 0.001
	*N*	926	926	926	926	926	926
birth weight (grams)	Pearson correlation	0.074*	−0.150**	−0.271**	1	0.399**	−0.318
	Sig. (2‐tailed)	0.024	< 0.001	< 0.001		< 0.001	< 0.001
	*N*	926	926	926	926	926	926
weight percentile	Pearson correlation	0.080*	0.059	−0.001	0.399**	1	−0.093
	Sig. (2‐tailed)	0.015	0.071	0.974	< 0.001		0.004
	*N*	926	926	926	926	926	926
postnatal age (days)	Pearson correlation	−0.027	0.215	0.330	−0.318	−0.093	1
	Sig. (2‐tailed)	0.405	< 0.001	< 0.001	< 0.001	0.004	
	*N*	926	926	926	926	926	926

** The correlation is significant at the 0.01 level (2‐tailed).

* The correlation is significant at the 0.05 level (2‐tailed).

## 4 Discussion

### 4.1 Interpretation

tcpCO_2_ is a routine non‐invasive parameter for the continuous monitoring of gas exchange in neonates. At present, tcpCO_2_ measurement cannot fully replace blood gas analyses but constitutes a useful and continuous trend parameter which substantially decreases the need for blood sampling. The correlation between tcpCO_2_ and pcCO_2_ found in this work is in good accordance with previous findings reported by Janaillac et al. [27], Aly et al. [32], and Borenstein‐Levin et al. [22], who reported correlation coefficients of tcpCO_2_ and pCO_2_ of approximately *r* = 0.6.

An improvement of pCO_2_ estimation from tcpCO_2_ has the potential to increase the time a patient spends within the pCO_2_ target range. This can consequently further reduce the number of required blood gas analyses and the rate of complications associated with pCO_2_ extremes. For the estimation of pCO_2_, different confounders have to be regarded.

Sandberg et al. previously defined the difference between tcpCO_2_ and paCO_2_ as the bias of tcpCO_2_ [21]. This research group found a higher bias in infants weighing less than 1 kg (4.5 ± 13.5 mmHg) and older than 7 days (5.3 ± 13.5 mmHg). However, the influence of the weight and postnatal age was only reported to be small. The data for the patient group of less than 1 kg are similar to those observed in our study in EPI. Although birth weight was associated with ΔpCO_2_ in our univariate analyses, this association was no longer significant after adjustment for gestational age, suggesting that gestational maturity rather than body weight per se accounted for the observed effect.

Another research group investigated the accuracy of tcpCO_2_ depending on venous pCO_2_ values [33]. They found a good agreement of tcpCO_2_ and venous pCO_2_ during episodes of hypocarbia and normocapnia (mean differences 1.5 ± 1.4 mmHg and 0 ± 2.3 mmHg). During hypercarbia, tcpCO_2_ was shown to be significantly lower than venous pCO_2_ (6.1 ± 8.9 mmHg). An important finding was the significant influence of pcCO_2_ levels on ΔpCO_2_. This observation is consistent with previous reports showing that transcutaneous CO_2_ monitoring accuracy decreases at higher pCO_2_ levels, whereas precision remains relatively stable [34]. The observed proportional bias likely reflects the physiological and technical limitations of transcutaneous measurements at elevated CO_2_ tensions, including altered skin perfusion and diffusion characteristics. Clinically, this suggests that tcpCO_2_ remains useful for continuous trend monitoring across a broad pCO_2_ range, but caution is warranted when interpreting absolute tcpCO_2_ values in patients with marked hypercapnia.

Our analysis showed that the gestational age might be another essential characteristic to be considered during tcpCO_2_ monitoring. We hypothesize that the differences between the groups originate from the thickness of the stratum corneum in the epidermis. Its number of cell layers is known to be dependent on gestational age [35]. The epidermis is considered mature at the gestational age of 34 weeks [36]. The facilitated CO_2_ diffusion through the immature skin may explain the overestimation of pcCO_2_ by tcpCO_2,_ particularly in more immature infants, and the significant differences in ΔpCO_2_ between EPI and the other groups. This systematic deviation might be overcome by the use of a correction factor. However, larger databases would be needed to make a valid suggestion for such a correction. Besides differences in epidermal structure, gestational age–related variations in hemodynamics and skin perfusion may also have contributed to the observed differences in measured pCO_2_ values. Since transcutaneous monitoring depends on adequate local capillary blood flow, altered peripheral perfusion could influence measurement characteristics. Previous studies have shown inverse correlations between gestational age and peripheral as well as cutaneous blood flow [37], indicating physiologic differences in skin perfusion between immature and more mature infants. However, because blood pressure and perfusion‐related parameters were unavailable in the present study, their potential influence could not be further evaluated.

For EPI and VPI, the skin maturation typically takes place faster than the chronological maturation of other organ systems [38]. In this work, we focused on the first postnatal week only to eliminate this potential influence. It would be interesting to evaluate the accuracy of tcpCO_2_ during the entire postnatal skin maturation process in EPI and VPI in the future.

### 4.2 Limitations

The temperature to which the tcpCO_2_ sensor is heated up is known to affect the deviation from pCO_2_ as it influences the perfusion of the skin. Our unit uses a temperature of only 40.2°C to minimize the risk of skin burns. We recognize that the choice of temperature may influence ΔpCO_2_. Previous studies demonstrated that the bias (ΔpCO_2_) decreases at higher operating temperatures and suggested the application of a bias correction in the case of operating temperatures below 42°C [39, 40].

Furthermore, tcpCO_2_ sensors are susceptible to sensor drift and shift [41, 42]. The sensor probes have to be replaced, recalibrated, and re‐membraned regularly [43]. Our database does not store information about this change in the location of the sensor or the perfusion of the skin, which can also have an impact on the quality of measurement. It was therefore not possible to consider these additional variables in the analysis. Because blood sampling was clinically driven rather than protocolized, the dataset may overrepresent periods of physiological instability or suspected monitoring inaccuracy. This could have biased agreement estimates and may limit generalizability to routine continuous monitoring conditions.

Blood gas analyses were conducted immediately after blood sampling. Within the window of tolerance of ±5 min, we extracted the corresponding tcpCO_2_ value. We recognize that even this small time span could introduce a bias and have an impact on the accuracy of tcpCO_2_.

Intensive care treatment was conducted according to local standards. The inclusion of the neonates into the study did not alter these standards and did not involve any other interventions. The local practices can potentially entail a center bias. We plan to validate our findings on external data to eliminate this influence.

As capillary blood sampling requires smaller amounts of blood [44] and avoids catheter‐associated complications, we predominantly use capillary blood gas analyses in our unit. We do not expect the source of blood sampling to have a substantial impact on our findings. Values for pcCO_2_ are marginally higher than arterial ones [45]. The directed effects which we have observed in this study would most likely have been more pronounced if arterial blood gas analyses had been used.

The heterogeneous requirements and pathophysiological conditions of premature and mature infants inevitably influence their critical care treatment [46]. The underlying pathology can, for example, have an impact on the number of blood gas analyses, which is deemed necessary by the medical staff. A substantial proportion of LPI and TI was admitted to the NICU due to transient tachypnea of the newborn, which led to only short observational periods and few blood gas analyses. Some of these infants had to be excluded from this analysis because tcpCO_2_ measurement was not conducted. In contrast, EPI and VPI typically require much longer stays on the NICU and almost always receive tcpCO_2_ monitoring. To minimize the resulting effect of unequal numbers of measurements per patient, we used an LMM.

### 4.3 Generalizability

This work uses observational data taken from a single‐center study and includes a total of 926 measurements from 116 neonatal patients. The dataset included a few EPIs (*n* = 22). Multi‐center analyses of larger cohorts and comparisons of different sites are required for improvement of generalizability. No a priori sample size estimation was performed because the study used an existing dataset, and the sample size was therefore fixed.

### 4.4 Conclusion

This is the first study to evaluate the effect of gestational age on the difference between tcpCO_2_ and pcCO_2_. We found a significant difference of ΔpCO_2_ depending on gestational age, EOBI, and pcCO_2_. This knowledge may be used to further improve the estimation of pCO_2_ from tcpCO_2_ in neonates in the future. At present, tcpCO_2_ monitoring is a useful additive diagnostic tool to regard pCO_2_ trends, while occasional blood gas analyses are still required.

## Author Contributions

**Lena Olivier:** conceptualization, data curation, formal analysis, investigation, methodology, visualization, writing – original draft preparation. **Camelia Lauterbach Oprea:** resources, software, validation, writing – review and editing. **André Stollenwerk:** resources, software, validation, writing – review and editing, funding acquisition, project administration. **Valerie Pfannschmidt:** validation, writing – review and editing. **Thorsten Orlikowsky:** resources, supervision, validation, writing – review and editing. **Mark Schoberer:** conceptualization, methodology, supervision, validation, writing – review and editing, funding acquisition, project administration.

## Ethics Statement

The study was approved by the local ethics committee (Ethik‐Kommission an der Medizinischen Fakultät der Rheinisch‐Westfälischen Technischen Hochschule Aachen [RWTH Aachen], approval number EK 24‐213). The study was performed in accordance with the Declaration of Helsinki [28].

## Consent

The authors have nothing to report.

## Conflicts of Interest

The authors declare no conflicts of interest.

## Data Availability

The data are available upon reasonable request from the corresponding author.

## References

[1] D. Braun , E. Braun and V. Chiu , “Trends in Neonatal Intensive Care Unit Utilization in a Large Integrated Health Care System,” JAMA Netw Open 3 (2020): e205239, 10.1001/jamanetworkopen.2020.5239. 32556257 PMC7303809

[2] W. Harrison and D. Goodman , “Epidemiologic Trends in Neonatal Intensive Care, 2007–2012,” JAMA Pediatr 169 (2015): 855–862, 10.1001/jamapediatrics.2015.1305. 26214387

[3] M. O. Edwards , S. J. Kotecha , and S. Kotecha , “Respiratory Distress of the Term Newborn Infant,” Paediatric Respiratory Reviews 14 (2013): 29–36; quiz 36‐27, 10.1016/j.prrv.2012.02.002. 23347658

[4] N. Holme and P. Chetcuti , “The Pathophysiology of Respiratory Distress Syndrome in Neonates,” Paediatrics and Child Health 22 (2012): 507–512, 10.1016/j.paed.2012.09.001.

[5] K. Khabbache , Y. Hennequin , D. Vermeylen , and B. Van Overmeire , “Current Respiratory Support Practices in Premature Infants: An Observational Study,” Pan African Medical Journal 39 (2021): 66, 10.11604/pamj.2021.39.66.14482. 34422189 PMC8363955

[6] C. R. Wheeler and C. D. Smallwood , “2019 Year in Review: Neonatal Respiratory Support,” Respiratory Care 65 (2020): 693–704, 10.4187/respcare.07720. 32209710

[7] A. Battisti‐Charbonney , J. Fisher , and J. Duffin , “The Cerebrovascular Response to Carbon Dioxide in Humans,” Journal of Physiology 589 (2011): 3039–3048, 10.1113/jphysiol.2011.206052. 21521758 PMC3139085

[8] S. Shankaran , J. Langer , S. Kazzi , et al., “Cumulative Index of Exposure to Hypocarbia and Hyperoxia as Risk Factors for Periventricular Leukomalacia in Low Birth Weight Infants,” Pediatrics 118 (2006): 1654–1659, 10.1542/peds.2005-2463. 17015558

[9] U. H. Thome and N. Ambalavanan , “Permissive Hypercapnia to Decrease Lung Injury in Ventilated Preterm Neonates,” Seminars in Fetal & Neonatal Medicine 14 (2009): 21–27, 10.1016/j.siny.2008.08.005. 18974027

[10] J. Fabres , W. A. Carlo , V. Phillips , G. Howard , and N. Ambalavanan , “Both Extremes of Arterial Carbon Dioxide Pressure and the Magnitude of Fluctuations in Arterial Carbon Dioxide Pressure are Associated With Severe Intraventricular Hemorrhage in Preterm Infants,” Pediatrics 119 (2007): 299–305, 10.1542/peds.2006-2434. 17272619

[11] M. Waitz , S. Nusser , M. Schmid , et al., “Risk Factors Associated With Intraventricular Hemorrhage in Preterm Infants With </= 28 Weeks Gestational Age,” Klinische Pädiatrie 228 (2016): 245–250, 10.1055/s-0042-111689. 27617760

[12] S. Bolisetty , A. Dhawan , M. Abdel‐Latif , et al., “Intraventricular Hemorrhage and Neurodevelopmental Outcomes in Extreme Preterm Infants,” Pediatrics 133 (2014): 55–62, 10.1542/peds.2013-0372. 24379238

[13] M. I. Lindinger and G. J. Heigenhauser , “Effects of Gas Exchange on Acid‐Base Balance,” Compr Physiol 2 (2012): 2203–2254, 10.1002/cphy.c100055. 23723036

[14] H. Balasubramanian , M. Bhanushali , V. Tripathi , et al., “Effect of Minimization of Early Blood Sampling Losses Among Extremely Premature Neonates: A Randomized Clinical Trial,” Journal of Pediatrics 269 (2024): 114002, 10.1016/j.jpeds.2024.114002. 38447757

[15] W. Hellstrom , L. Forssell , E. Morsing , K. Savman , and D. Ley , “Neonatal Clinical Blood Sampling Led to Major Blood Loss and Was Associated With Bronchopulmonary Dysplasia,” Acta Paediatrica 109 (2020): 679–687, 10.1111/apa.15003. 31505053 PMC7155086

[16] M. Rudiger , K. Topfer , H. Hammer , G. Schmalisch , and R. R. Wauer , “A Survey of Transcutaneous Blood Gas Monitoring Among European Neonatal Intensive Care Units,” BMC Pediatrics 5 (2005): 30, 10.1186/1471-2431-5-30. 16092957 PMC1192805

[17] W. Mindt , P. Eberhard , and R. Schäfer , “Monitoring of PCO2 by Skin Surface Sensors,” Biotelemetry and Patient Monitoring 9 (1982): 28–35. 6809070

[18] S. Mukhopadhyay , R. Maurer , and K. M. Puopolo , “Neonatal Transcutaneous Carbon Dioxide Monitoring‐‐Effect on Clinical Management and Outcomes,” Respiratory Care 61 (2016): 90–97, 10.4187/respcare.04212. 26508771

[19] D. R. Murdoch and B. A. Darlow , “Handling During Neonatal Intensive Care,” Archives of Disease in Childhood 59 (1984): 957–961, 10.1136/adc.59.10.957. 6497433 PMC1628870

[20] A. Conway , E. Tipton , W. Liu , et al., “Accuracy and Precision of Transcutaneous Carbon Dioxide Monitoring: A Systematic Review and Meta‐Analysis,” Thorax 74 (2019): 157–163, 10.1136/thoraxjnl-2017-211466. 30209079

[21] K. L. Sandberg , H. Brynjarsson , and O. Hjalmarson , “Transcutaneous Blood Gas Monitoring During Neonatal Intensive Care,” Acta Paediatrica 100 (2011): 676–679, 10.1111/j.1651-2227.2011.02164.x. 21244487

[22] L. Borenstein‐Levin , N. Avishay , O. Soffer , et al., “Transcutaneous CO(2) Monitoring in Extremely Low Birth Weight Premature Infants,” Journal of Clinical Medicine 12 (2023): 5757, 10.3390/jcm12175757. 37685823 PMC10488371

[23] P. Baumann , V. Gotta , S. Adzikah , and V. Bernet , “Accuracy of a Novel Transcutaneous PCO2 and PO2 Sensor With Optical PO2 Measurement in Neonatal Intensive Care: A Single‐Centre Prospective Clinical Trial,” Neonatology 119 (2022): 230–237, 10.1159/000521809. 35124680

[24] N. Binder , H. Atherton , T. Thorkelsson , and S. B. Hoath , “Measurement of Transcutaneous Carbon Dioxide in Low Birthweight Infants During the First Two Weeks of Life,” American Journal of Perinatology 11 (1994): 237–241, 10.1055/s-2008-1040754. 8048993

[25] K. Bendjelid , N. Schütz , M. Stotz , et al., “Transcutaneous PCO2 Monitoring in Critically Ill Adults: Clinical Evaluation of a New Sensor,” Critical Care Medicine 33 (2005): 2203–2206, 10.1097/01.ccm.0000181734.26070.26. 16215371

[26] L. L. Aliwalas , L. Noble , K. Nesbitt , et al., “Agreement of Carbon Dioxide Levels Measured by Arterial, Transcutaneous and End Tidal Methods in Preterm Infants < or = 28 Weeks Gestation,” Journal of Perinatology 25 (2005): 26–29, 10.1038/sj.jp.7211202. 15496874

[27] M. Janaillac , S. Labarinas , R. E. Pfister , and O. Karam , “Accuracy of Transcutaneous Carbon Dioxide Measurement in Premature Infants,” Critical Care Research and Practice 2016 (2016): 8041967, 10.1155/2016/8041967. 27375901 PMC4916268

[28] World Medical Association , “Declaration of Helsinki: Ethical Principles for Medical Research Involving Human Subjects,” Journal of the American Medical Association 310 (2013): 2191–2194, 10.1001/jama.2013.281053. 24141714

[29] E. von Elm , D. Altman , M. Egger , et al., “The Strengthening the Reporting of Observational Studies in Epidemiology (STROBE) Statement: Guidelines for Reporting Observational Studies,” Journal of Clinical Epidemiology 61 (2008): 344–349, 10.1016/j.jclinepi.2007.11.008. 18313558

[30] J. Lawn . Born too soon: The Global Action Report on Preterm Birth. 2012.

[31] L. E. Scott , J. S. Galpin , and D. K. Glencross , “Multiple Method Comparison: Statistical Model Using Percentage Similarity,” Cytometry. Part B, Clinical Cytometry 54 (2003): 46–53, 10.1002/cyto.b.10016. 12827667

[32] S. Aly , M. El‐Dib , M. Mohamed , and H. Aly , “Transcutaneous Carbon Dioxide Monitoring With Reduced‐Temperature Probes in Very Low Birth Weight Infants,” American Journal of Perinatology 34 (2017): 480–485, 10.1055/s-0036-1593352. 27673754

[33] S. Uslu , A. Bulbul , M. Dursun , et al., “Agreement of Mixed Venous Carbon Dioxide Tension (PvCO2) and Transcutaneous Carbon Dioxide (PtCO2) Measurements in Ventilated Infants,” Iranian Journal of Pediatrics 25 (2015): e184, 10.5812/ijp.184. 26199686 PMC4505968

[34] R. J. Martin , A. Beoglos , M. Miller , et al., “Increasing Arterial Carbon Dioxide Tension: Influence on Transcutaneous Carbon Dioxide Tension Measurements,” Pediatrics 81 (1988): 684–687. 3128769

[35] P. Cartlidge , “The Epidermal Barrier,” Seminars in Neonatology 5 (2000): 273–280, 10.1053/siny.2000.0013. 11032710

[36] Y. N. Kalia , L. B. Nonato , C. H. Lund , and R. H. Guy , “Development of Skin Barrier Function in Premature Infants,” Journal of Investigative Dermatology 111 (1998): 320–326, 10.1046/j.1523-1747.1998.00289.x. 9699737

[37] P. Y. Wu , W. Wong , G. Guerra , et al., “Peripheral Blood Flow in the Neonate; 1. Changes in Total, Skin, and Muscle Blood Flow With Gestational and Postnatal Age,” Pediatric Research 14 (1980): 1374–1378, 10.1203/00006450-198012000-00023. 7208156

[38] Y. B. Chiou and U. Blume‐Peytavi , “Stratum Corneum Maturation,” A Review of Neonatal Skin Function. Skin Pharmacol Physiol 17 (2004): 57–66, 10.1159/000076015. 14976382

[39] J. M. Rennie , “Transcutaneous Carbon Dioxide Monitoring,” Archives of Disease in Childhood 65 (1990): 345–346, 10.1136/adc.65.4_spec_no.345. 2110802 PMC1590151

[40] K. Hirata , M. Nishihara , Y. Oshima , S. Hirano , and H. Kitajima , “Application of Transcutaneous Carbon Dioxide Tension Monitoring With Low Electrode Temperatures in Premature Infants in the Early Postnatal Period,” American Journal of Perinatology 31 (2014): 435–440, 10.1055/s-0033-1352485. 23918520

[41] S. K.‐w Ng , E. Y.‐t Chan , and S.‐y Leung , “Comparison of Calibration Drift in Transcutaneous Carbon Dioxide Monitoring Devices for Overnight Level 4 Sleep Study in Hong Kong Children,” Pediatric Respirology and Critical Care Medicine 8 (2024): 25–32, 10.4103/prcm.prcm_21_23.

[42] W. van Weteringen , T. Goos , T. van Essen , et al., “Novel Transcutaneous Sensor Combining Optical tcPO(2) and Electrochemical tcPCO(2) Monitoring With Reflectance Pulse Oximetry,” Medical and Biological Engineering and Computing 58 (2020): 239–247, 10.1007/s11517-019-02067-x. 31741291 PMC6994448

[43] P. Eberhard , “The Design, Use, and Results of Transcutaneous Carbon Dioxide Analysis: Current and Future Directions,” Anesthesia and Analgesia 105 (2007): S48–S52, 10.1213/01.ane.0000278642.16117.f8. 18048898

[44] J. L. Krleza , A. Dorotic , A. Grzunov , et al., “Capillary Blood Sampling: National Recommendations on Behalf of the Croatian Society of Medical Biochemistry and Laboratory Medicine,” Biochem Med (Zagreb) 25 (2015): 335–358, 10.11613/BM.2015.034. 26524965 PMC4622200

[45] A. M. Harrison , J. M. Lynch , J. M. Dean , and M. K. Witte , “Comparison of Simultaneously Obtained Arterial and Capillary Blood Gases in Pediatric Intensive Care Unit Patients,” Critical Care Medicine 25 (1997): 1904–1908, 10.1097/00003246-199711000-00032. 9366777

[46] A. A. Chakkarapani , C. Roehr , S. Hooper , et al., “Transitional Circulation and Hemodynamic Monitoring in Newborn Infants,” Pediatric Research 96 (2024): 595–603, 10.1038/s41390-022-02427-8. 36593283 PMC11499276

